# Slow Dynamics of Ring Polymer Melts by Asymmetric Interaction of Threading Configuration: Monte Carlo Study of a Dynamically Constrained Lattice Model

**DOI:** 10.3390/polym11030516

**Published:** 2019-03-19

**Authors:** Eunsang Lee, YounJoon Jung

**Affiliations:** Department of Chemistry, Seoul National University, Seoul 08826, Korea; napsang324@gmail.com

**Keywords:** ring polymer, threading dynamics, topological glass, lattice model, Monte Carlo simulation

## Abstract

Abnormally slower diffusional processes than its internal structure relaxation have been observed in ring polymeric melt systems recently. A key structural feature in ring polymer melts is topological constraints which allow rings to assume a threading configuration in the melt phase. In this work, we constructed a lattice model under the assumption of asymmetric diffusivity between two threading rings, and investigated a link between the structural correlation and its dynamic behavior via Monte Carlo simulations. We discovered that the hierarchical threading configurations render the whole system to exhibit abnormally slow dynamics. By analyzing statistical distributions of timescales of threading configurations, we found that the decoupling between internal structure relaxation and diffusion is crucial to understand the threading effects on the dynamics of a ring melt. In particular, in the limit of small but threaded rings, scaling exponents of the diffusion coefficient *D* and timescale $\tau_{\mathrm{diff}}$ with respect to the degree of polymerization *N* agree well with that of the annealed tree model as well as our mean-field analysis. As *N* increases, however, the ring diffusion abruptly slows down to the glassy behavior, which is supported by a breakdown of the Stokes–Einstein relation.

## 1. Introduction

Topological interactions of nonconcatenated and unknotted ring polymers have provided many challenging questions to polymer scientists in recent years [1,2,3,4,5,6,7,8,9,10,11,12,13,14,15,16,17,18,19]. The lack of free chain end makes the phase space of the ring melt different from that of a linear polymer melt. This implies that the structural and dynamical properties of the ring polymer cannot be explained by either a simple random walk or a self-avoiding walk model. Instead, there have been many theoretical and simulation studies made by utilizing such topological interactions to elucidate the structure of the ring, and some of these studies provide evidence for isolated globular structure in the asymptotic limit [1,2,3,4,5,20].

In addition to the structure, dynamical and rheological properties of the rings are shown to be distinct from those of linear counterparts. Recent experimental studies have succeeded in synthesizing monodisperse ring polymers with high purity, and those works have revealed an abnormally slow dynamical regime observed even without any plateau modulus in shear stress experiments [6,7,8,9,10,11,12]. A great effort to elucidate the ring melt dynamics has also been made with aids of theoretical and simulation models [1,3,14,15,16,17,18,19,21,22]. Many of these works tried to predict the scaling exponent δ of diffusion constant *D* with respect to its degree of polymerization *N*, $D∼N^{-\delta }$ for the ring polymer melts. For example, in the very early stage, δ=2 for a ring diffusing in fixed obstacles was predicted based on a lattice tree model [1,14], δ=1.9 for a ring melt on an annealed tree model [21], and δ=5/3 on a self-similar scaling model for a fractal loopy globule with a fractal dimension 3 [19]. Molecular dynamics (MD) simulation is also a useful tool to numerically evaluate δ from a microscopic point of view, e.g. Tsolou et al. reported δ between 1.1 and 1.3 for polyethylene rings whose *N* is shorter than the entanglement length, $N_{e}$ [23]. Halverson et al. found even larger δ=2.4 for long rings of $N>N_{e}$ using a bead–spring model and also supported the view that slow molecular diffusion is decoupled from its internal structure relaxation [17]. Most recently, Tsalikis et al. published experimental and simulation results showing δ=2 for long ring melts of polyethylene oxide [24]. While these studies provide useful information on the dynamical properties of a ring melt in individual system, it seems still unclear how much and to what extent the ring dynamics are affected by the topological constraints, due to, first, the theoretical difficulty in modeling the topological constraint in a proper way, and, second, a challenge involved with long time simulation studies. In this regard, it would be absolutely useful and necessary to develop a theoretical model that can cover all the emerging characteristics of the ring dynamics that result from the topological constraints. Due to the very nature of the topological constraint itself, however, it seems highly non-trivial to develop such a theory.

The physical origin for dynamic behaviors of the ring has been suggested in a few previous studies [16,18,25,26,27,28]. In these studies, importance of a specific configuration between two rings, so-called *threading*, has been proposed. In our previous work based on MD simulations of a ring polymer bead-spring model [16], we found that the threading configuration makes it very slow for the ring polymers to diffuse away freely, which is verified by the long time tail of contact time distributions. This observation results in the decoupling between persistence and exchange time distributions for molecular contacts of two rings. Mathematical definitions of threading configuration based on the method of minimal surfaces [27] and Contour Reduction Topological Analysis (CReTA ) algorithm [28] also support the threading scenario, such that threading mainly controls the long-time relaxation modes. By means of Monte Carlo simulation, Lo et al. tried to understand the threading effect on the slow ring dynamics based on the assumption of one dimensional diffusion for unthreading [29].

Interestingly, there have also been a few efforts to understand the dynamical behavior of the rings in terms of topological glass [29,30,31]. Sakaue et al. recently published a theoretical work for glassy dynamics of a dense ring solution by a concept of cooperative motion of many neighboring rings [31]. Michieletto et al. also reported a heterogeneous dynamics of dense ring polymer solutions under the topological constraint of random pinning perturbations [22]. However, microscopic evidence for the glassy and heterogeneous dynamics of the ring is still insufficient. In this paper, therefore, we elucidate the ring polymer dynamics depending on the asymmetric interaction of two rings and their cooperative motion which may contribute to understand the nature of topological glass and its transition.

In this regard, we consider how threading configuration between rings slows down ring polymer dynamics by constructing a coarse-grained lattice model. This model is developed under the assumption of asymmetric diffusivity of two inter-threaded rings and shows how this assumption affects the slow and glassy dynamics of the ring polymer melts.The rest of this paper is organized as follows. In Section 2, we explain physical idea behind our model that can well mimic threading dynamics of a ring polymer as well as the simulation methods. Mean-field analysis and Monte Carlo simulation results of our model are provided in Section 3. Concluding remarks follow in Section 4.

## 2. Model and Simulation Methods

We develop a coarse-grained lattice model that can describe the intermolecular correlation between rings using a set of rules based on the kinetic constraints. We provide both analytical and numerical results from the model to gain physical insights on how the threading between two rings induces its glassy dynamics. The major problem of studying ring polymer physics often stems from the failure in dealing with the topological interactions in a proper way. One of the issues in this respect is to find an appropriate length scale of interchain correlation for the ring which corresponds to an entanglement length for the linear polymer case [17,32]. Although a theoretical model for the presence of such a length scale has not been fully established yet due to the difficulty in incorporating the inherent topological interaction of the ring in the model, several numerical studies have supported the existence of such a length scale. In these studies, small rings are proven to behave as a Rouse chain only with even Rouse modes [23] and a crossover from Gaussian to non-Gaussian behavior with the increase of a ring size is shown [11,16,17,19,33,34]. MD simulation studies also indicate that the crossover length weakly depends on the polymer molecular weight [2,17]. Therefore, it is reasonable to start our discussion of ring polymer dynamics by separating out length and timescales into two regimes, in much the same way as the linear polymer melt employs the concept of entanglement time and length scales.

We now call the length scale of the crossover as a threading length, $N_{\mathrm{th}}$, which has also been called an entanglement length for ring melts in previous works [17,19,20,21]. We assume that intermolecular interaction of the threading exists beyond the length scale of a threading blob size. An internal structure below this length scale is assumed to be relaxed by the annealed tree model done by Grosberg et al. [20,21]. In this model, a ring polymer configuration can be mapped on an annealed tree whose backbone length is *L*. The backbone is self-avoiding in space, so the size of the ring is scaled as $R∼L^{\nu_{F}}$ where $\nu_{F}=0.588$. A ring in an asymptotic limit has a compact globular structure of $R∼N^{\nu }$ with ν=1/3, and *L* is also scaled as $L∼N^{\rho }$ with $\rho =1/3\nu_{F}=0.567$. A chain relaxation takes place by the diffusion of threading (entanglement) blobs along the chain backbone. The whole chain is relaxed if the backbone moves by *L* in a time scale of $\tau_{l}∼\tau_{0}{\left(N/N_{\mathrm{th}}\right)}^{\rho +2}$ where $\tau_{0}$ is Rouse time of an threading blob depending on $N_{\mathrm{th}}$. Without considering the threading effect, the self diffusion coefficient of the chain center of mass is given by the $R^{2}/\tau_{l}∼{\left(N/N_{\mathrm{th}}\right)}^{2\nu -\rho -2}$. However, if the threading configuration exists between two rings, the diffusion of entanglement blobs is restricted, which has to be handled in a different way.

**Figure 1:**
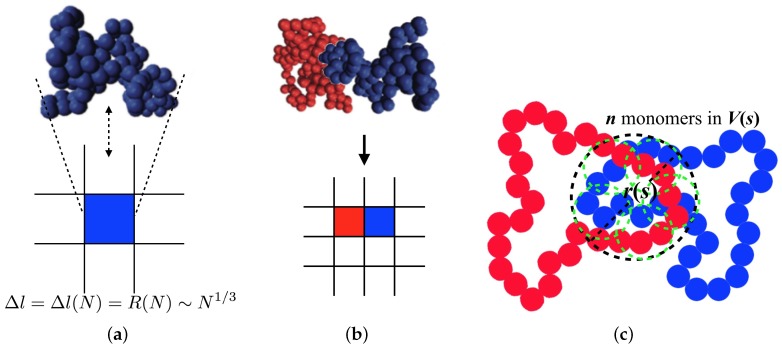
(**a**,**b**) Schematic description of our lattice model. One lattice describes one ring polymer and the unit size of the lattice is, therefore, a function of degree of polymerization, *N*. This figure shows how we mimic the asymmetric diffusivity of two threading rings. (**c**) A description for shared volume between two rings. Green circles represent threading blobs, each of which contains $N_{\mathrm{th}}$ monomers.

To implement the threading into the model described above, we introduce a coarse-grained lattice model (Figure 1a,b). On a square lattice, each ring polymer is represented by an individual lattice site. We deal with the ring polymers whose lengths are longer than $N_{\mathrm{th}}$ based on the idea that, as the longer the length of the ring is, the more the threading configuration affects its dynamics. We implicitly incorporate *N* and the relaxation time scale of the non-threading ring of the length *N* into the lattice constant and a Monte Carlo step. At first, we should express a lattice constant, Δl, in terms of *N* to see *N*-dependent dynamic properties of ring polymers. Obviously, Δl=R since a ring occupies one lattice site. In the annealed tree model, a ring of $N\geq N_{\mathrm{th}}$ shows a size scaled as $R∼N^{1/3}$, thus the lattice constant is given by:

(1) $$\Delta l=R\left(N\right)\approx bN^{1/3}.$$

It is also very important to renormalize a unit time (a Monte Carlo step), Δt, in terms of *N*. Here, Δt corresponds to how long it takes for the non-threading ring polymer to move to the next site. As mentioned above, the annealed tree model without considering threading predicts the chain relaxation time as $\tau_{l}$, thus the unit time scale can be given by:

(2) $$\Delta t=\tau_{l}\approx cN^{\rho +2}.$$

In Equations (1) and (2), *b* and *c* are coefficients depending on system properties, $N_{\mathrm{th}}$, temperature, density, and friction coefficient of a monomer, not depending on *N*.

Based on the scaling relations of Δt and Δl on *N*, we now implement a certain type of dynamical rules to consider the effect of the threading configuration. This method is motivated by a kinetically constrained model which has been originally utilized to model glassy systems and their heterogeneous dynamics [35,36,37]. We develop our model under a main assumption of asymmetric diffusivity of two rings in a threading configuration. Threading and threaded rings are expected to have different average structures, e.g., radius of gyration. Considering a threaded ring as a fixed obstacle, a threading ring has its size smaller than a certain length scale until unthreaded. In contrast, a threaded ring cannot collapse with the size smaller than the certain length scale until unthreaded. We expect that such a difference causes an asymmetric diffusivity of two rings and we study in this work the extreme asymmetric case that while a threading ring (blue) can diffuse away, a movement of a threaded ring (red) is completely restricted, as shown in Figure 1b. Diffusion of the threaded ring is recovered if the threading one diffuses away or if they relax their internal structure to escape from a restrained configuration. The asymmetric diffusivity has not actually been proven either theoretically or numerically, so this assumption should be strictly revisited in the future. Nevertheless, this model can be informative to understand how the asymmetric diffusivity of two contacting molecules affects the system dynamics. Such a local dynamic rule can be implemented in our model by considering a pair variable $c_{ij}$ between a nearest neighbor *i* and *j*, such that

(3) $$c_{ij}=\left\{\begin{array}{c} 0,\;\;\;\;\mathrm{non}\text{-}\mathrm{threading}\;\mathrm{configuration},\\ 1,\;\;\;\;\mathrm{passive}\;\mathrm{threading}:\;i\mathrm{th}\;\mathrm{molecule}\;\mathrm{is}\;\\ \;\;\;\;\;\;\;\mathrm{threaded}\;\mathrm{by}\;j\mathrm{th},\;\\ -1,\;\;\mathrm{active}\;\mathrm{threading}:\;i\mathrm{th}\;\mathrm{molecule}\;\mathrm{is}\\ \;\;\;\;\;\;\;\mathrm{threading}\;j\mathrm{th}.\; \end{array}\right.$$

For example, if the *i*th molecule threads the *j*th, $c_{ij}=-1$ and $c_{ji}=1$. The diffusivity of the *i*th molecule is affected by the intermolecular correlation between the *i*th molecule and the neighboring *j*th molecules:

(4) $$m_{i}={\prod_{j}}^{\prime }\left(1-\delta_{1,c_{ij}}\right)=\left\{\begin{array}{c} 0,\;\;\;\;\mathrm{immobile},\\ 1,\;\;\;\;\mathrm{mobile}, \end{array}\right.$$

where the prime indicates multiplication only for the nearest neighbors of the *i*th, and $\delta_{ij}$ is a Kronecker delta. This equation implies that, if at least one pair property among its neighbors are passive, the *i*th molecule is immobile, otherwise mobile.

**Figure 2:**
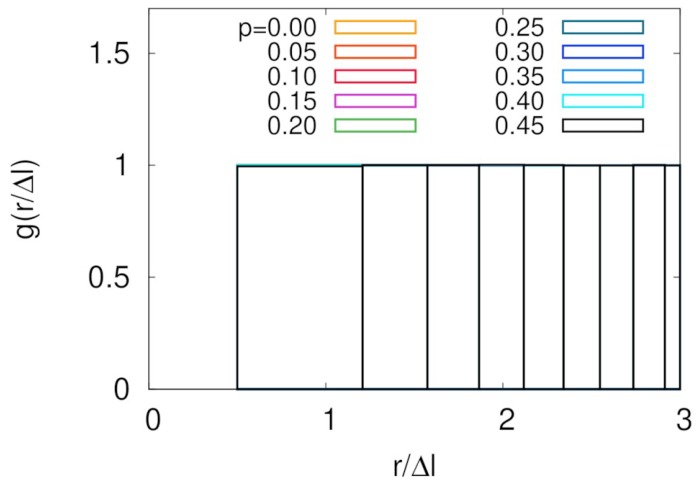
Radial distribution functions for different threading probabilities shown in different colors. All graphs with different *p*s overlap with each other.

Simulation on our lattice model was performed as follows. Since there is no structural correlation except for excluded volume interactions, initial equilibrium configuration is easily achieved by self-avoiding, random arrangements of particles on the lattice. After locating all the particles, pair variables are randomly assigned with the probability *p*, that is, the probability for the pair to be in a threading configuration, for all the pairs. Mobility for each particle is determined by Equation (4). A trial move of a randomly chosen particle to a nearest neighbor position is accepted only if the following three conditions are satisfied: the particle is mobile in an old configuration, the proposed new position is empty, and the particle at the proposed position is mobile, as determined by $c_{ij}$s with its new neighbors. It is physically obvious to check the particle’s old mobility and the particle exclusion. The last condition is related to maintaining the detailed balance, which is discussed in the following paragraph. In both cases of accepted and rejected trial moves, the chosen particle undergoes an intramolecular structural relaxation process by reassigning $c_{ij}$ with all its neighbors. It should be noticed that the average number of contacting molecules remains constant regardless of *N* shown in previous MD simulation study [2]. This condition is automatically achieved by our lattice-based simulation with a fixed space dimension and particle fraction regardless of *p*. We set the system dimension to d=5 and particle fraction to f=0.8, which leads to the number of average neighboring molecules of $\bar{z}=2df=8$. Such choice of the system parameters allowed us not only to describe well the threading dynamics but also to sample efficiently the dynamic properties of the ring with a reasonable acceptance ratio of about 0.2. Although we used dynamical interaction only with nearest neighbors, the higher space dimension in this model than in experiments allowed describing a non-spherical shape of a ring that can form a highly probable dynamical interaction in larger length scale than its average size. Moreover, our model can describe a molecule that is both threading and threaded with the others using the large number of average neighboring molecules, which is also frequently observed in the all-atom simulation [2]. For comparison, we also performed simulations for the system with the real space dimension of d=3 (f=0.5, $\bar{z}=3$), and the difference is briefly discussed below. The system size is L=6, which is proven to be large enough to ignore finite size effect due to the lack of structural correlation in our model (see Figure 2). We performed standard Monte Carlo (MC) simulation with the threading probabilities, *p*, from 0.00 to 0.45 by an interval of 0.05. In total, $10^{7}$ MC steps were used to take an equilibrium ensemble average. It is not so obvious how to take dynamical properties from MC simulation, but a detailed mapping of real time scale on one MC sweep mentioned above made it possible to extract meaningful dynamical properties of ring polymer melts.

To prove the detailed balance of this model, imagine a transition from a state *a* to *b*. The transition rate $w_{a\to b}$ is $P_{\mathrm{mob}}\left(a\right)P_{\mathrm{exc}}\left(b\right)P_{\mathrm{mob}}\left(b\right)$ where $P_{\mathrm{mob}}\left(a\right)$ is a probability that a chosen particle is mobile in the state *a* and $P_{\mathrm{exc}}\left(b\right)$ is a probability of the proposed position being empty which is proportional only to a particle density. Therefore, $w_{a\to b}=w_{b\to a}$ for any states *a* and *b*. In this model, all microstates in equilibrium are equally probable due to no intermolecular interaction except for the particle exclusion. Figures S1 and S2 demonstrate that three- and two-particle test systems with d=2, L=6 and *p* = 0.3 satisfy the detailed balance. In Figure S1, the microstate of the three-particle system can be defined by $x_{s}=\left\{{\vec{x}}_{12},{\vec{x}}_{23}\right\}$ where ${\vec{x}}_{12}={\vec{x}}_{1}-{\vec{x}}_{2}$, and all the microstates in equilibrium were equally probable except the states of ${\vec{x}}_{12}=0$, ${\vec{x}}_{23}=0$, and ${\vec{x}}_{12}={\vec{x}}_{23}$ due to the particle exclusion. In Figure S2, probability of the microstates given by $P(v_{1},v_{2})$, where ${\vec{x}}_{12}=\left(v_{1},v_{2}\right)$, also shows that all the possible microstates were equally visited during our simulation, clearly proving the detailed-balance was satisfied.

It is essential to take the *N*-dependence of the particle mobility into account in order to study the dynamic scaling law of ring polymer melts. We take *p*, an average probability of $c_{ij}$ having unity when *i*th and *j*th molecules are neighboring to each other, as the main control parameter in our simulation study. This probability is interpreted as a threading probability between a molecular pair in real polymeric systems. To find the *N*-dependence of this thread-forming probability, p(N), we envisioned that, when two rings are close in contact, they share a volume of size *r*, including *s* monomers of each ring shown in Figure 1c. The shared region is composed of $n_{\mathrm{th}}=s/N_{\mathrm{th}}$ threading blobs, each of which has $N_{\mathrm{th}}$ monomers, as shown in Figure 1c. This blob is passively threaded by the blob belonging to the different ring with the probability $p_{\mathrm{th}}$, which depends on the chemical details of a polymer and density but not on the degree of polymerization. Since at least one passive threading between blobs makes the ring be in a dynamically constrained (threaded) state, a probability of not forming a passively threaded configuration between two contacting rings is given by a mean-field approximation as a multiplication of non-threading probabilities for all threading blobs in the shared volume, that is ${\left(1-p_{\mathrm{th}}\right)}^{n_{\mathrm{th}}}$. The threading probability of two contacting ring is, therefore, $p\left(N\right)\approx 1-{\left(1-p_{\mathrm{th}}\right)}^{n_{\mathrm{th}}}$. To find the *N*-dependence of $n_{\mathrm{th}}$, we needed to consider the number of monomers in the shared region. It was proven by an MD simulation study that the number of surface monomers contacting with other rings is scaled as $∼N^{\eta ≃0.95}$, and the number of neighboring rings is constant regardless of *N* also for the asymptotic rings [2]. Therefore, *s* is also scaled as $N^{\eta }$ and the number of threading blobs can be approximated by $n_{\mathrm{th}}=s/N_{\mathrm{th}}\approx a\;N^{\eta }$. The final approximate relation of p(N) is given by:

(5) $$p\left(N\right)\approx 1-{\left(1-p_{\mathrm{th}}\right)}^{aN^{\eta }},$$

where *a* is a coefficient independent of *N*. It should be noticed that, for the small value of $ap_{\mathrm{th}}N^{\eta }$, p(N) is approximated to $p\left(N\right)\approx ap_{\mathrm{th}}N^{\eta }∼N^{\eta }$.

## 3. Results and Discussions

We first show radial distribution functions of our system in Figure 2. Due to the high space dimension and entailing large memory cost during simulation, linear dimension of our model was restricted to the relatively small value of L=6. Nevertheless, the structure shown in this figure guaranteed that there was no structural correlation except for zero distance. The reason is obvious because we implemented only dynamic rules including particle exclusion without any additional interaction potential between particles. This result strongly supports that our simulation box was large enough to ignore any finite size effect. Additional evidence for negligible finite size effect in our model is provided in the later part of this section.

Before showing simulation results for dynamic properties, we calculated dynamic properties using a mean-field approach. The average probability for the particle to be mobile is expressed by ${\bar{p}}_{m}\left(p\right)={\left(1-p\right)}^{\bar{z}}$ because all $c_{ij}$ should not be passive for all neighbors. The average diffusion time necessary for the particle to move the distance of its size is expressed by the following equation:

(6) $$\begin{array}{ccc} \tau_{\mathrm{diff}}\left(p\right) & \approx & {\bar{p}}_{m}\left(p\right)\frac{\Delta t}{1-f}+\left(1-{\bar{p}}_{m}\left(p\right)\right)\frac{1}{{\bar{p}}_{m}\left(p\right)}\frac{\Delta t}{1-f}\\ & \approx & \frac{p_{m}{\left(p\right)}^{2}-p_{m}\left(p\right)+1}{\left(1-f\right)p_{m}\left(p\right)}\Delta t. \end{array}$$

In the first line of this equation, the first term of right hand side represents diffusion time of a mobile particle and the second term is for an immobile particle. Mobility of the mobile particle is determined not only by a fraction of unoccupied neighboring sites but also that of the immobile one requiring a transition time to be mobile, which is taken into account by the factor $1/{\bar{p}}_{m}$. Moreover, the diffusion coefficient of the ring can be obtained from the lattice constant, the diffusion time, and the spatial dimension in the following way:

(7) $$D\left(p\right)=\frac{\Delta l^{2}}{2d\tau_{\mathrm{diff}}}\approx \frac{\Delta l^{2}}{2d\Delta t}{\left[\frac{p_{m}{\left(p\right)}^{2}-p_{m}\left(p\right)+1}{\left(1-f\right)p_{m}\left(p\right)}\right]}^{-1}$$

It is important to note that Δl, Δt and *p* are all *N*-dependent values, which are discussed in detail below.

**Figure 3:**
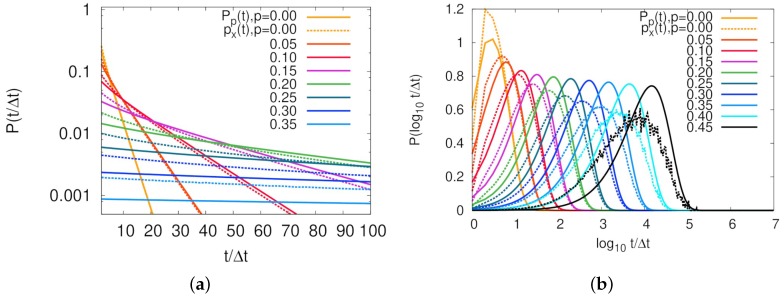
Decoupling of persistence ($p_{p}\left(t\right)$, solid lines) and exchange ($p_{x}\left(t\right)$, dashed lines) time distributions for molecular contacts: (**a**) a linear-log plot that clearly shows an exponential decay of both $p_{p}\left(t\right)$ and $p_{x}\left(t\right)$ for p=0; and (**b**) $P(\log_{10}t)$ versus $\log_{10}t$ showing that, the larger is *p*, the more clearly shown is the decoupling of two distributions. Different colors represent different average threading probabilities, *p*.

We now show how our model well describes the dynamic properties of ring polymers, especially by focusing on the threading feature. As mentioned before, our previous work [16] showed phenomenological evidence for the threading dynamics, which is a decoupling phenomenon of persistence and exchange time distributions for the molecular contact. Here, we calculated the same property using a similar definition of the molecular contact in our lattice model. We first defined the contact of the molecular pair such that it has value 1 if two molecules are in the neighboring sites, or 0 otherwise. Using this variable, the persistence time $\tau_{p}$ was defined by the time duration over which the molecular contact persists from any time point of a contact at $t=t_{0}$ to the loss of the contact at $t=t_{0}+\tau_{p}$. The exchange time, $\tau_{x}$, is a contact lifetime, differently defined as a time duration from contact-creating at $t=t_{c}$ to contact-loss at $t=t_{c}+\tau_{x}$. Distributions of these two time scales are related by $P_{p}\left(t\right)=\int_{t}^{\infty }dt^{\prime }P_{x}\left(t^{\prime }\right)/\langle \tau_{x}\rangle$, where $P_{p}\left(t\right)$ and $P_{x}\left(t\right)$ are distributions of persistence and exchange times, respectively. $\langle \tau_{x}\rangle$ is an average exchange time calculated by $\int_{0}^{\infty }dtP_{x}\left(t\right)t$. Figure 3 represents persistence and exchange time distributions of our model for various *p*’s. For a purely Poissonian process, it is well known that $P_{x}\left(t\right)$ is exponential, and identical to $P_{p}\left(t\right)$ [16,37]. The case of p=0 mimics such a situation, as clearly shown in linear-log plot of the distributions in Figure 3a. Molecular contact between two particles was Poissonian and time scale was exactly Δt/(1−f), as estimated by the first term in Equation (6). For the case of non-zero threading probabilities, Figure 3b shows that, as *p* increased, long tails in exchange time distribution induced much longer average persistence times than average exchange times. Although this result cannot be directly compared to the MD simulation result [16] due to the difficulty of mapping *p* on *N* with the knowledge of $N_{\mathrm{th}}$ and other parameters, the fact that decoupling between two time distributions is observed in both simulations provides strong evidence of our lattice model well capturing the central feature of the threading dynamics.

**Figure 4:**
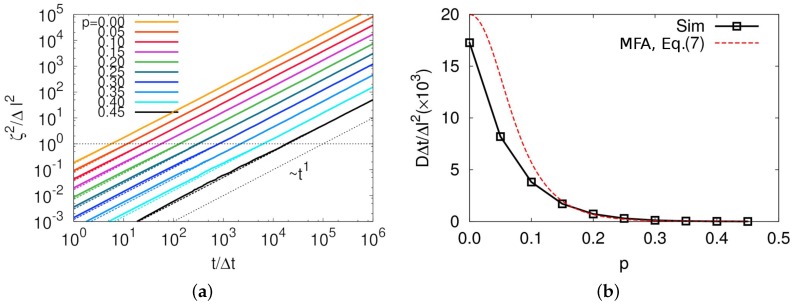
(**a**) Mean square displacements as a function of t/Δt for various threading probabilities, *p*s; and (**b**) diffusion coefficients versus *p*. The diffusion coefficient in (**b**) was obtained by fitting MSDs as a function of *t* in diffusive regimes to $D=\zeta^{2}/2dt$. In (**a**), all the fitted functions are shown by dashed lines and a black dotted line represents $\zeta^{2}∼t^{1}$. In (**b**), squares are the simulation results (Sim) and a red dashed line represents Equation (7) from mean-field approximation (MFA).

We show self-diffusion coefficients of the particle from MC simulation, which corresponds to the diffusion coefficient of a ring center of mass to compare the results with the analytical result in Equation (7). To do so, we first calculated mean square displacements $\zeta^{2}\left(t\right)$ for various values of *p*, which clearly exhibited a slowing down of diffusion with increasing *p*, as shown in Figure 4a. The ballistic motion of polymer monomers was, of course, hidden below the timescale of Δt, which cannot be shown in this model. Moreover, a very weak sub-diffusive motion in ζ<Δl regime was observed for all of the *p* values, except for p=0 case. Even at p=0.45, an exponent γ≈0.92 in the relation $\zeta^{2}∼t^{\gamma }$ was observed, which was found to be 3/4 in the previous MD simulation [17]. We believe that this weak sub-diffusive motion originated from the strongly simplified diffusion mechanism not considering molecular shape in this model. Figure 4b shows diffusion coefficients obtained by fitting MSD to $\zeta^{2}\left(t\right)=2Ddt$ in the diffusive regime of γ=1 where *d* is space dimension. Both length and time have their own dimension, thus resulting that the diffusion coefficient also has a unit of $\Delta l^{2}/\Delta t$, as shown in Figure 4b.

**Figure 5:**
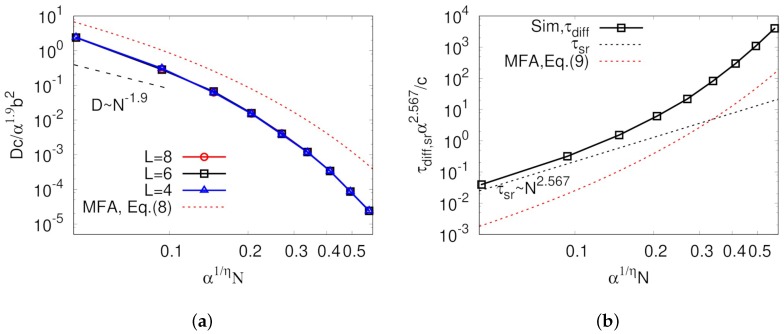
(**a**) Diffusion coefficients; and (**b**) diffusion times as a function of degree of polymerization, *N*. In (**a**), red circles, black squares and blue triangles represent diffusion coefficients obtained from different system size, L=8, 6, and 4, respectively. In (**b**), simulation result of L=6 is depicted by square symbols. In both figures, black and red dotted lines represent Equations (8) and (9), respectively, with ρ=0.567 and η=0.95. Blue dashed lines indicate the approximate scaling relation in our small αN simulation regime, such that $D∼N^{-\rho -4/3}$ and $\tau_{\mathrm{diff}}∼N^{\rho +2}$, respectively. In both graphs, α, *b*, and *c* are numerical constants to scale the threading probability unit time and unit length on *N*, respectively.

As mentioned above, it is very important to keep in mind that all unit time, unit length, and threading probability are functions of *N*, which allowed representing the diffusion coefficients as a function *N* as well as finding scaling relations that have been usually studied in polymer physics. Using the relations in Equations (1), (2), and (5), we can rewrite Equations (7) and (6) such that:

(8) $$\begin{array}{ccc} D(N) & \approx & \frac{\Delta l^{2}}{2d\Delta t}{\left[\frac{p_{m}{\left(N\right)}^{2}-p_{m}\left(N\right)+1}{\left(1-f\right)p_{m}\left(N\right)}\right]}^{-1}\\ & \approx & \frac{1}{2d}{\left[\frac{e^{-2\bar{z}\alpha N^{\eta }}-e^{-\bar{z}\alpha N^{\eta }}+1}{\left(1-f\right)e^{-\bar{z}\alpha N^{\eta }}}\right]}^{-1}\frac{b^{2}}{c}N^{-\rho -4/3}, \end{array}$$

and

(9) $$\begin{array}{ccc} \tau_{\mathrm{diff}}\left(N\right) & \approx & \frac{p_{m}{\left(N\right)}^{2}-p_{m}\left(N\right)+1}{\left(1-f\right)p_{m}\left(N\right)}\Delta t\\ & \approx & \frac{e^{-2\bar{z}\alpha N^{\eta }}-e^{-\bar{z}\alpha N^{\eta }}+1}{\left(1-f\right)e^{-\bar{z}\alpha N^{\eta }}}cN^{\rho +2}, \end{array}$$

where $\alpha \equiv -a\ln(1-p_{\mathrm{th}})$ and $\bar{z}=2df$. Normalized diffusion coefficients using Equation (8) are shown in Figure 5a. Over the entire range of *p* values studied, obtained diffusion coefficients from the simulation were smaller than expected from the mean-field in Equation (8). A dashed line in Figure 5a shows that, for the small *N* case, the decreasing rate of diffusion coefficient seemed to be δ≈2.0>1.9, as observed in many simulation studies [16,17,34,38]. In Equation (8), *D* starts decreasing with the rate of $N^{-\rho -4/3≃1.9}$ if $\bar{z}\alpha N^{\eta }$ (or ${\left(\bar{z}\alpha \right)}^{1/\eta }N$) is close to zero, but in Figure 5a this regime is hidden below the lowest $\alpha N^{\eta }$ value of our simulation. This increasing δ with increasing *N* was also observed in the MD simulation work done by Brown et al. [33]. It is quite important to keep in mind that *N* is still larger than $N_{\mathrm{th}}$ even for the ring in the limit of small *N* in this analysis because our model considers only rings in a threading regime. Here, α is small because $p_{\mathrm{th}}\ll 1$, which makes $\bar{z}\alpha N^{\eta }$ small enough even for relatively large *N*. As the degree of polymerization further increases, the diffusion coefficient decreases more quickly with δ>3, which has not been observed in previous MD simulation studies. This rapid decrease of the diffusion coefficient is even faster than expected by mean-field. This is believed to be glassy dynamics originated from highly immobile large clusters, which is discussed below. Figure 5a also shows that our result does not depend on the system size from L=4–8, which supports that our system size was sufficiently large to observe reasonable scaling relation in dynamic properties for our model.

Another interesting phenomenon observed in our study is decoupling of diffusion time and intramolecular structure relaxation time, which is one of the well-known properties of ring dynamics [16,17]. Figure 5b shows the comparison between the diffusion and intramolecular structure relaxation times as a function of *N*. The diffusion time was calculated by the average time taken for the particle to move around to the nearest neighboring site. In addition to the diffusion coefficient, the diffusion time in Equation (9) can be reduced to $\tau_{\mathrm{diff}}∼N^{\rho +2≃2.567}$ for small $\bar{z}\alpha N^{\eta }$. As *N* increases, diffusion time more rapidly increases with the higher exponent than for small $\bar{z}\alpha N^{\eta }$. According to the definition of the unit time designed in our model, internal structure relaxation occurrs in every Δt, leading to the relaxation time being scaled by $\tau_{\mathrm{sr}}∼N^{2.567}$; thus, these two timescales are more largely decoupled when *N* is larger. Such decoupling between internal structure relaxation and diffusion times strongly supports that the dynamic correlation originated from the asymmetric interaction of two contacting rings is the main reason for abnormal ring dynamics.

Of course, any information about α, *b*, and *c*, which include the dependence of the macroscopic properties on system parameters other than *N*, e.g., density, temperature, and chemical details of polymers, could not be calculated in this simulation. However, it is highly expected that all the simulated and experimental results of the diffusion coefficients and the diffusion times merge in master curves in Figure 5 if they are normalized by proper parameters such as α, *b*, and *c*. There have been a few efforts to elucidate the relation between the polymer size and *b* for an asymptotic ring [32,39], but others for dynamic properties have been completely unknown. Therefore, convincing evidence for these parameters by theoretical and numerical studies should be provided in the future. Nevertheless, this finding is meaningful because we implemented the microscopic view of the dynamical and structural properties of the ring melt to the mesoscopic simulation model by the mean-field approximation, and we obtained the macroscopic scaling behavior by simulating the model.

**Figure 6:**
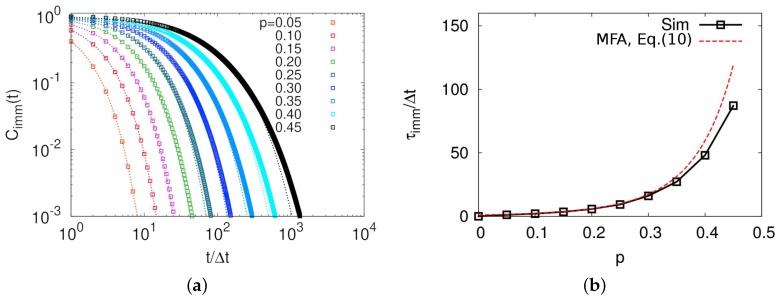
(**a**) Time-correlation functions of particle immobility for various values of *p*s; and (**b**) immobility relaxation times as a function of *p*. Open squares in both graphs represent simulation results and the red dotted line in (**b**) indicates $\Delta t/{\left(1-p\right)}^{\bar{z}}$.

For both diffusion coefficients and diffusion times, while their scaling dependence on *N* for small value of *N* was in a good agreement with mean-field predictions, the system became much slower than expected from the theory as *p* approaches to 0.5. We found that Equation (6) well describes the local mobility by observing a time correlation function of the particle immobility, as shown in Figure 6. The correlation function is defined by $C_{\mathrm{imm}}\left(t\right)=\left(\langle \left(1-m_{i}\left(0\right)\right)\cdot \left(1-m_{i}\left(t\right)\right)\rangle -{\left(1-\langle m\rangle \right)}^{2}\right)/\langle {\left(1-m\right)}^{2}\rangle$ where 〈⋯〉 indicates an ensemble average. The correlation times for the particle immobility, $\tau_{\mathrm{imm}}$, obtained by integrating the correlation function, indicates how long the immobility of the particle persists over time. Figure 6b shows that calculated $\tau_{\mathrm{imm}}$s from simulations were almost identical to those expected from the mean-field approach,

(10) $$\begin{array}{c} \tau_{\mathrm{imm}}=\Delta t/{\left(1-p\right)}^{\bar{z}}. \end{array}$$

This is because the dynamics in our model follows a Markovian rule, in which intermolecular correlation becomes memoryless beyond $\tau_{\mathrm{sr}}$. Nevertheless, slower dynamics of our model in large *p* regime than the mean-field prediction can be explained by the correlation on longer length and time scales, which was also supported by the multiple threading configuration of rings by MD simulations [18,28]. For example, imagine a ring $R_{1}$ is threaded by a ring $R_{2}$, which is again threaded by a ring $R_{3}$. In the high threading probability regime (large *N*) and very low probability of the particle to be mobile by the internal structure relaxation, the ring $R_{1}$ becomes mobile if it is unthreaded by $R_{2}$, which also happens if $R_{2}$ is unthreaded by $R_{3}$. Such a multi-ring effect makes a huge cluster of immobile particles and makes the system dynamics really slow. Therefore, we expect that the dynamical correlation in a length scale of a large immobile cluster results in the slow dynamics, which can be an evidence of the topological glass.

**Figure 7:**
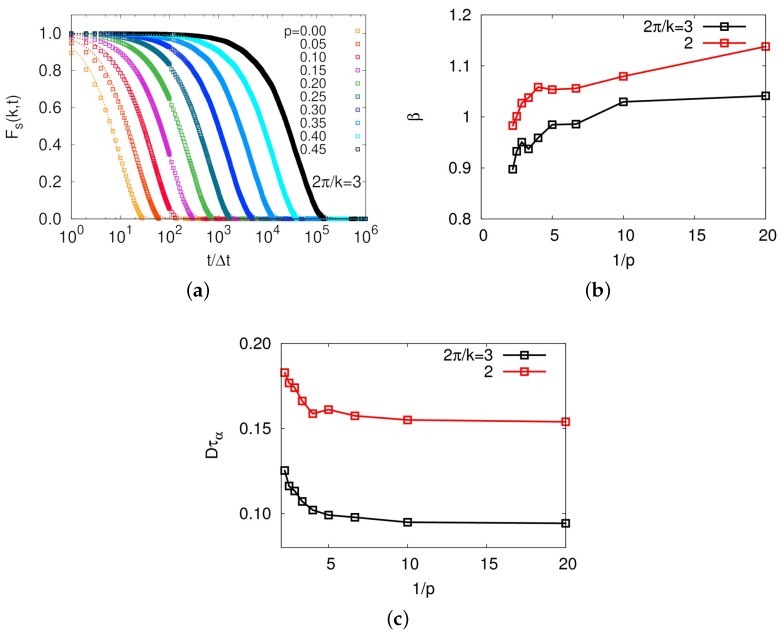
(**a**) Self-part of intermediate scattering functions with k=2π/3 for different values of *p*s; (**b**) exponents of the fitted stretched exponential functions of intermediate scattering functions; and (**c**) $D\tau_{\alpha }$ as a function of 1/p. Black and red lines indicate results with k=2π/3 and π, respectively. In (**b**,**c**), decreasing exponent and increasing $D\tau_{\alpha }$ with increasing *p* are shown, which is strong evidence for heterogeneous dynamics.

A strong dynamical correlation is usually manifested by a breakdown of Stokes–Einstein relation, which has been intensively studied in numerous glassy systems [36,37,40,41,42,43]. While studying dynamics of molecular center-of-mass of a ring in a melt with MD simulations is quite time-consuming, this model allowed us to readily obtain evidence for heterogeneous dynamics. We first calculated a self-part of intermediate scattering function defined by $F_{s}\left(k,t\right)=\langle \int dr\exp\left[-\mathrm{ik}\cdot (r_{i}\left(t\right)-r_{i}\left(0\right))\right]\rangle$, as shown in Figure 7a. Intermediate scattering functions were well fitted by stretched exponential, $\tilde{F_{s}}\left(k,t\right)=\exp\left[-{\left(t/\tau_{\alpha }\right)}^{\beta }\right]$, except for the case of p=0, and the exponent decreased below unity with increasing *p*, indicating heterogeneous dynamics for large *p* systems, as shown in Figure 7b. Consistent behavior was also evident in the violation of Stoke-Einstein relation, as shown in Figure 7c. This behavior well agrees with the previous argument that the discrepancy of diffusion times between simulation results and theoretical prediction for large *p* systems is originated from the dynamical correlation. All these observations strongly support that rings in a melt belong to a topological glass [29,30], since the intrinsic topology of connected chain end forms a specific conformation called threading, which directly leads to glassy dynamics.

Figure S3 describes the diffusion coefficient and the diffusion time for the system in the experimental dimension d=3 (f=0.5 and L=30). In both properties, slopes from the simulation and the mean-field approach were consistent with each other, which did not happen in d=5 system. In the lattice-based model, space dimension of d=3 allowed the maximum number of neighbors to be only 6. The probability of the particle being mobile $p_{m}={\left(1-p\right)}^{\bar{z}}$ rapidly decreased with increasing *p* when the number of neighboring rings was large. In such a small fraction of mobile particle, the probability of forming a big immobile cluster was also high, which caused abnormally slow diffusion of the particles. Therefore, although we have mainly discussed the results from the system in the unrealistic space dimension d=5, we expect that a large $\bar{z}$ value, which is similar with the MD simulation result, enables well describing the realistic intermolecular correlation.

## 4. Conclusions

We propose a simple model system to describe dynamics of ring polymers in a melt. The model is nothing more complicated than incorporating asymmetric dynamics states between two threading rings originated from the topological effect of rings, but still it well explains various dynamical properties of ring polymer melts. In particular, a long time tail of molecular contact time distribution indicates that the dynamic asymmetricity of two contacting molecules can strongly slow down the molecule diffusion. Both the diffusion coefficient *D* and timescale $\tau_{\mathrm{diff}}$ first exhibit scaling behaviors of the annealed tree model, $D∼N^{-1.9}$ and $\tau_{\mathrm{diff}}∼N^{2.567}$, and then later turn into more abrupt behaviors as the threading probability increases with *N*. Our approximate mean-field results well describe the behavior qualitatively for small *N* regime, but the dynamics of large *N* system is even slower than expected from the mean-field due to the formation of dynamically constrained clusters. Such a dynamic correlation induces a significant breakdown of Stokes–Einstein relation, which can be taken as strong evidence for the topological glass. Our model is highly simplified to describe a threading configuration between two rings, therefore no information below the threading length and time scales can be provided. Furthermore, the model is built on the assumption of asymmetric diffusivity of threading rings, which has to be tested. Nevertheless, the fact that the asymmetric diffusivity leads to enormous slowing down of ring diffusion and glassy dynamics can provide us a physical insight of understanding the ring dynamics in combination with the polymer topology and glassy dynamics.

## Supplementary Materials

The following are available online at https://www.mdpi.com/2073-4360/11/3/516/s1, Figure S1: Probability of microstates in equilibrium for two-dimensional three-particle system with L=6. Figure S2: Contour plot of probability of microstates $x_{12}=\left(v_{1},v_{2}\right)$ in equilibrium for two-dimensional two-particle system with L=6. Figure S3: Mean square displacement versus *t*, diffusion coefficients and diffusion times as a function of degree of polymerization, *N*, for the system of d=3, f=0.5.
