# Supplementary material for: Slow Dynamics of Ring Polymer Melts by Asymmetric Interaction of Threading Configuration: Monte Carlo Study of a Dynamically Constrained Lattice Model
Source: Polymers (Basel). 2019 Mar 19;11(3):516. doi: 10.3390/polym11030516 (PMC6473489; doi:10.3390/polym11030516)
Supplement: Supplementary file 1 [file polymers-11-00516-s001.pdf]

Supplementary Materials: THREADING DYNAMICS OF RING POLYMER MELTS REVEALED BY DYNAMICALLY CONSTRAINED LATTICE MODEL

Eunsang Lee† and YounJoon Jung*

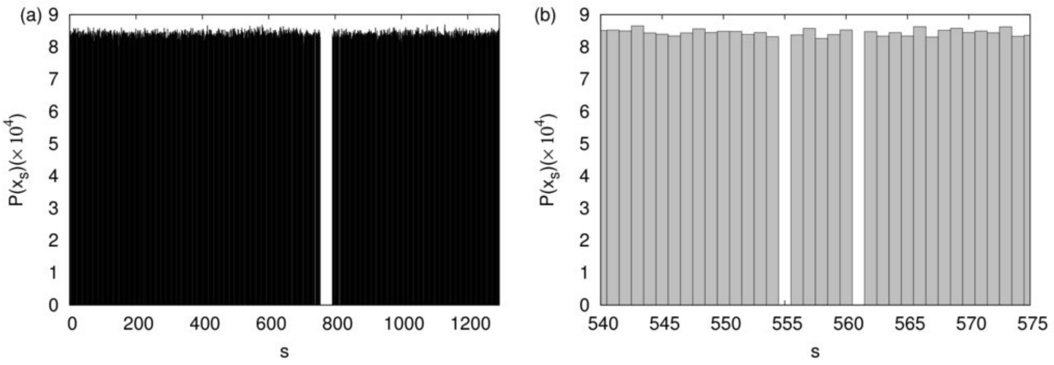

Figure S1. Probability of microstates $\mathbf{x}_s = (v_1, v_2, w_1, w_2)$ in equilibrium for two-dimensional three-particle system with $L = 6$, where $\vec{x}_{12} = (v_1, v_2)$ and $\vec{x}_{13} = (w_1, w_2)$. The state index s is defined by $s = (v_1 + L_h)L^3 + (v_2 + L_h)L^2 + (w_1 + L_h)L + w_2 + L_h$ where $L_h = L/2$. (a) shows the probability of whole states, and (b) shows only for the states with $\vec{v}_{12} = (-1, 0)$. In (a), $756 \le s \le 791$ where $P(\mathbf{x}_s) = 0$ represents the state of $\vec{v}_{12} = (0, 0)$, which is not allowed by the particle exclusion. In (b) the states of the probability zero in this graph are for $\vec{w} = (-1, 0)$ and $\vec{w} = (0, 0)$. Except for the case of the particle exclusion, probabilities of all states are almost the same which demonstrates the validity of the detailed balance.

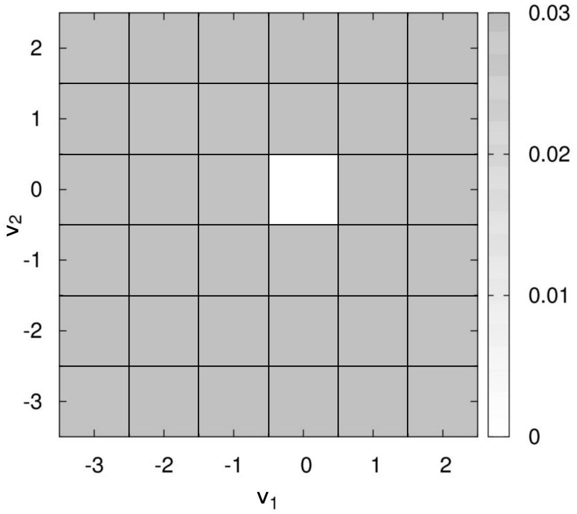

Figure S2. Contour plot of probability of microstates $x_{12} = (v_1, v_2)$ in equilibrium for two-dimensional two-particle system with $L = 6$. Every state except $\vec{x}_{12} = (0, 0)$ is equally probable.

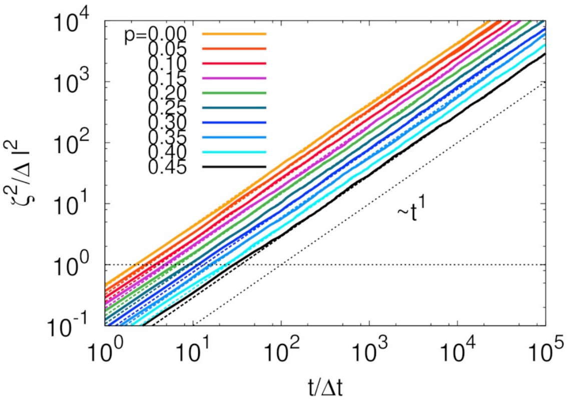

(a)

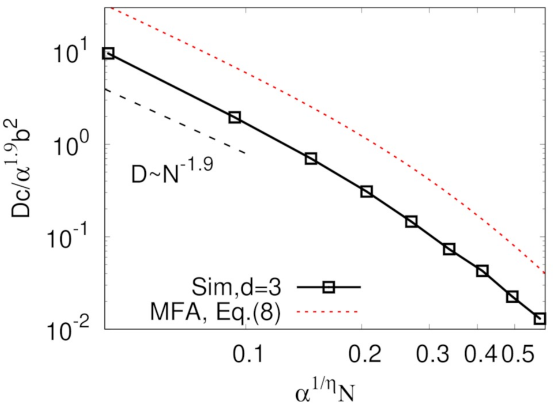

(b)

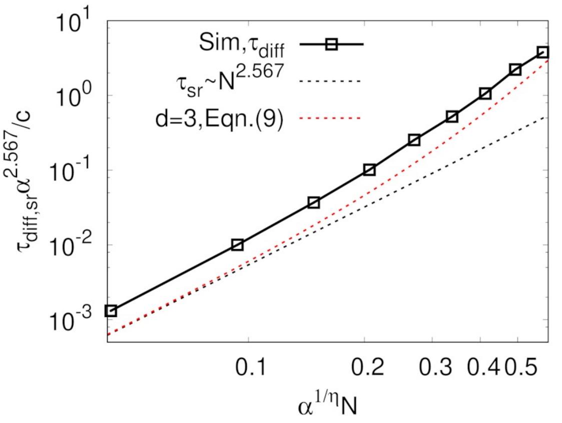

(c)

Figure S3. (a) Mean square displacement versus t, (b) diffusion coefficients and (c) diffusion times as a function of degree of polymerization, N for the system of $d = 3$, $f = 0.5$. In Figs.(b) and (c), red dotted lines represent Eqns. (8) and (9) in the main text, respectively. Black dashed lines indicate the approximate scaling relations in small αN regime, such that $D \sim N^{-1.9}$ and $\tau_{\rm diff} \sim N^{2.567}$, respectively. In both graphs, α, b, and c are numerical constants to scale the threading probability unit time and unit length on N, respectively.
